# Effects of WIN 55,212-2 (a non-selective cannabinoid CB_1_ and CB_2_ receptor agonist) on the protective action of various classical antiepileptic drugs in the mouse 6 Hz psychomotor seizure model

**DOI:** 10.1007/s00702-014-1173-7

**Published:** 2014-02-19

**Authors:** Magdalena Florek-Luszczki, Aleksandra Wlaz, Maria W. Kondrat-Wrobel, Piotr Tutka, Jarogniew J. Luszczki

**Affiliations:** 1 Department of Public Health, Institute of Rural Health, Jaczewskiego 2, 20-950 Lublin, Poland; 2 Department of Pathophysiology, Medical University, Ceramiczna 1, 20-150 Lublin, Poland; 3 Department of Pharmacology, Institute of Nursing and Health Sciences, University of Rzeszow, Warzywna 1, 35-959 Rzeszow, Poland; 4 Isobolographic Analysis Laboratory, Institute of Rural Health, Jaczewskiego 2, 20-950 Lublin, Poland; 5 Department of Pathophysiology, Medical University, Jaczewskiego 8, 20-090 Lublin, Poland

**Keywords:** 6 Hz psychomotor seizure model, Antiepileptic drugs, Cannabinoids, Drug interactions, WIN 55,212-2

## Abstract

The aim of this study was to characterize the influence of WIN 55,212-2 (WIN—a non-selective cannabinoid CB_1_ and CB_2_ receptor agonist) on the anticonvulsant effects of various classical antiepileptic drugs (clobazam, clonazepam, phenobarbital and valproate) in the mouse 6 Hz-induced psychomotor seizure model. Limbic (psychomotor) seizure activity was evoked in albino Swiss mice by a current (32 mA, 6 Hz, 3 s stimulus duration) delivered via ocular electrodes. Drug-related adverse effects were ascertained by use of the chimney test (evaluating motor performance), step-through passive avoidance task (assessing learning) and grip-strength test (evaluating skeletal muscular strength). Total brain concentrations of antiepileptic drugs were measured by fluorescence polarization immunoassay to ascertain any pharmacokinetic contribution to the observed antiseizure effect. Results indicate that WIN (5 mg/kg, administered intraperitoneally) significantly enhanced the anticonvulsant action of clonazepam (*P* < 0.001), phenobarbital (*P* < 0.05) and valproate (*P* < 0.05), but not that of clobazam in the mouse 6 Hz model. Moreover, WIN (2.5 mg/kg) significantly potentiated the anticonvulsant action of clonazepam (*P* < 0.01), but not that of clobazam, phenobarbital or valproate in the 6 Hz test in mice. None of the investigated combinations of WIN with antiepileptic drugs was associated with any concurrent adverse effects with regard to motor performance, learning or muscular strength. Pharmacokinetic experiments revealed that WIN had no impact on total brain concentrations of antiepileptic drugs in mice. These preclinical data would suggest that WIN in combination with clonazepam, phenobarbital and valproate is associated with beneficial anticonvulsant pharmacodynamic interactions in the mouse 6 Hz-induced psychomotor seizure test.

## Introduction

Accumulating experimental evidence indicates that one of the synthetic cannabimimetic compounds [i.e., WIN 55,212-2 mesylate (WIN)—a highly potent non-selective cannabinoid CB_1_ and CB_2_ receptor agonist] potentiated the anticonvulsant activity of some classical antiepileptic drugs (i.e., diazepam, carbamazepine, phenytoin, phenobarbital and valproate) and second-generation antiepileptic drugs (i.e., lamotrigine, pregabalin and topiramate) in the mouse maximal electroshock-induced tonic seizure (MES) model (Luszczki et al. 2011b, 2013; Naderi et al. 2008). WIN also enhanced the anticonvulsant activity of ethosuximide, phenobarbital and valproate in the mouse pentylenetetrazole-induced clonic seizure (PTZ) model (Luszczki et al. 2011a).

Considering the above-mentioned facts, it was of importance to continue experiments and determine the influence of WIN on the anticonvulsant action of some classical antiepileptic drugs (i.e., clonazepam, clobazam, phenobarbital and valproate) in the mouse 6 Hz-induced psychomotor seizure model. Low-frequency (6 Hz), long-duration (3 s) electrical stimulation in mice produces seizures characterized by immobility, focal clonus, and automatic behaviors reminiscent of human limbic epilepsy (Barton et al. 2001). Noteworthy, in this experimental model, one can readily assess the anticonvulsant potential of agents and compounds possessing the anticonvulsant properties, as well as determine their effects on antiepileptic drugs, effective in suppressing limbic seizures in humans (Barton et al. 2001).

We sought, therefore, to determine whether WIN would enhance the protective action of the selected classical antiepileptic drugs against limbic seizures in the mouse 6 Hz-induced psychomotor seizure model. Additionally, to determine the acute adverse-effect profiles for the combinations of WIN with clonazepam, clobazam, phenobarbital and valproate, the chimney test (a measure of motor performance impairment), the step-through passive avoidance task (a measure of learning deficits), and the grip-strength test (a measure of skeletal muscular strength impairment) were used. Finally, total brain antiepileptic drug concentrations were measured with fluorescence polarization immunoassay to ascertain whether any observed significant effects were consequent to a pharmacodynamic and/or a pharmacokinetic interaction.

## Materials and methods

### Animals and experimental conditions

All experiments were performed on adult male Swiss mice weighing 22–26 g. The animals were purchased from a licensed breeder (J. Kolacz, Warszawa, Poland). The mice were kept in colony cages with free access to food (chow pellets, Agropol S.J., Motycz, Poland), and tap water under standardized housing conditions (natural light–dark cycle, temperature of 21 ± 1 °C, relative humidity of 55 ± 5 %). After 7 days of adaptation to laboratory conditions, the animals were randomly assigned to experimental groups consisting of eight mice per group. Each mouse was used only once. All tests were performed between 9.00 and 15.00. Procedures involving animals and their care were conducted in conformity with current European Communities Council Directive of 24 November 1986 (86/609/EEC) and Polish legislation on animal experimentation. Additionally, all efforts were made to minimize animal suffering and to use only the number of animals necessary to produce reliable scientific data. The experimental protocols and procedures listed were approved by the Second Local Ethics Committee at the University of Life Sciences in Lublin (License Nos.: 86/2009, 24/2011, 17/2012) and conformed with the *Guide for the Care and Use of Laboratory Animals*.

### Drug administration

The following drugs were used in this study: clobazam (Frisium^®^, Sanofi-Aventis Deutschland GmbH, Frankfurt am Main, Germany), clonazepam (Polfa, Warszawa, Poland), phenobarbital (Polfa, Kraków, Poland), valproate (magnesium salt—kindly donated by ICN-Polfa S.A., Rzeszów, Poland), and WIN ((R)-(+)-[2,3-dihydro-5-methyl-3-(4-morpholinylmethyl)-pyrrolo-[1,2,3-de]-1,4-benzoxazin-6-yl]-1-naphthalenylmethanone mesylate; Tocris Bioscience, Bristol, UK). All drugs, except for valproate and WIN, were suspended in a 1 % solution of Tween 80 (Sigma-Aldrich, St. Louis, MO, USA) in distilled water, while valproate and WIN were dissolved in distilled water only. All drugs were administered intraperitoneally (i.p.) as a single injection in a volume of 5 ml/kg body weight. Fresh drug solutions were prepared on each day of experimentation and administered as follows: clonazepam—15 min, WIN—20 min, clobazam and valproate—30 min and phenobarbital—60 min before initiation of psychomotor seizures evoked by 6 Hz corneal electrical stimulation, evaluation of motor coordination, grip-strength and learning tests, as well as before brain sampling for the measurement of antiepileptic drug concentrations. The pretreatment times before testing of the antiepileptic drugs were based on information about their biological activity from the literature and our previous experiments (Luszczki et al. 2009, 2010). The times to the peak of maximum anticonvulsant effects for all antiepileptic drugs were used as the reference times in all behavioral tests and pharmacokinetic estimation of total brain antiepileptic drug concentrations. The route of i.p. administration of WIN and the pretreatment time before testing of its anticonvulsant effect were based on information from previous experiments (Naderi et al. 2008). The control animals received an equivalent volume of vehicle (1 % Tween 80).

### The Six-Hertz (6 Hz) psychomotor seizure model

Psychomotor (limbic) seizures were induced via corneal stimulation (6 Hz, 0.2 ms rectangular pulse width, 32 mA, 3 s duration) delivered by an S48 Square Pulse Stimulator and CCU1 Constant Current Unit (Grass Technologies, West Warwick, RI, USA). Ocular anesthetic (0.5 % solution of tetracaine hydrochloride) was applied to the mouse corneas 1 min before stimulation. Animals were manually restrained and released immediately following the stimulation and observed for the presence or absence of seizure activity. Before stimulation, the corneal electrodes were wetted with saline to provide good electrical contact. Immediately following stimulation, mice were placed separately in Plexiglas cages (25 × 15 × 10 cm) for behavioral observation. Following the stimulation, the animals exhibited a “stunned” posture associated with rearing and automatic movements that lasted from 60 to 120 s in untreated animals. The low-frequency (6 Hz) long-duration (3 s) seizures were characterized by immobility or stun, jaw and forelimb clonus, twitching of the vibrissae, and an elevated tail or Straub-tail (Barton et al. 2001; Brown et al. 1953). Animals resumed their normal exploratory behavior after the seizure. The experimental endpoint was protected against the seizure: an animal was considered to be protected if it resumed its normal exploratory behavior within 10 s after stimulation. Protection in the 6 Hz model was defined as the absence of a seizure. Mice not experiencing seizures exhibited normal exploratory behavior when placed in the cages (Brown et al. 1953). In the present study, to determine median effective doses (ED_50_ values) of antiepileptic drugs, the drugs were administered i.p. at the following dose ranges: clobazam, 0.5–4 mg/kg; clonazepam, 0.001–0.05 mg/kg; phenobarbital, 4–20 mg/kg; and valproate, 50–150 mg/kg. These antiepileptic drug doses suppressed psychomotor seizures in 10–90 % of mice subjected to the 6 Hz psychomotor seizure test. Using the log-probit method, the median effective doses (ED_50_ values) were determined using a minimum of eight mice per dose (Litchfield and Wilcoxon 1949), after which mice were euthanized by CO_2_ narcosis.

### Measurement of total brain antiepileptic drug concentrations

Pharmacokinetic evaluation of total brain antiepileptic drug concentrations was performed only for those combinations of WIN with antiepileptic drugs, whose anticonvulsant effect in the 6 Hz test was significantly greater than that for control (an antiepileptic drug + vehicle-treated) animals. Thus, the measurement of total brain concentrations of clonazepam, phenobarbital and valproate was undertaken at the doses, which corresponded to their ED_50_ values from the 6 Hz-induced psychomotor seizure test. Mice were killed by decapitation at times reflecting the peak of maximum anticonvulsant effects for the drugs in the 6 Hz psychomotor test. The whole brains of mice were removed from skulls, weighed, harvested and homogenized using Abbott buffer (1:2 wt/vol) in an Ultra-Turrax T8 homogenizer (IKA Werke, Staufen, Germany). The homogenates were centrifuged at 10,000*g* for 10 min The supernatant samples (75 μl) were analyzed by fluorescence polarization immunoassay for clonazepam, phenobarbital and valproate content using a TDx analyzer and reagents exactly as described by the manufacturer (Abbott Laboratories, North Chicago, IL, USA). For the quantitation of clonazepam, the benzodiazepine assay kit was used. The detection limit for benzodiazepine concentration in the TDx analyzer was 12 ng/ml. Thus, the analytical technique employed to quantify clonazepam concentrations at a dose of 0.0022 mg/kg was not sensitive enough to detect clonazepam concentrations; therefore, the drug was evaluated at a dose of 2.2 mg/kg (i.e., 1,000-fold higher). Total brain antiepileptic drug concentrations are expressed in μg/ml (except for clonazepam, whose concentrations were expressed in ng/ml) of brain supernatants as mean ± SEM of at least eight separate brain preparations.

### Step-through passive avoidance task

The effects of WIN, classical antiepileptic drugs and their combinations, at the ED_50_ values from the mouse 6 Hz test on learning in mice were quantified by the step-through passive avoidance task of Venault et al. (1986). On the first day before training, each animal was administered WIN, clonazepam, clobazam, phenobarbital, and valproate either singly or in combination at doses corresponding to their ED_50_ values from the 6 Hz test. The time before the commencement of the training session (after drug administration) was identical to that for the 6 Hz model. Subsequently, animals were placed in an illuminated box (10 × 13 × 15 cm) connected to a larger dark box (25 × 20 × 15 cm) equipped with an electric grid floor. Entrance of animals to the dark box was punished by an adequate electric footshock (0.6 mA for 2 s). The animals that did not enter the dark compartment were excluded from subsequent experimentation (overall, 5 % of animals used in this test) and replaced with those that correctly performed the task. On the following day (24 h later), the pre-trained animals were placed again into the illuminated box and observed up to 180 s. Mice that avoided the dark compartment for 180 s were considered to remember the task. The time that the mice took to enter the dark box was noted and the median latencies (retention times) with 25th and 75th percentiles were calculated. The step-through passive avoidance task gives information about ability to acquire the task (learning) and to recall the task (retrieval). Therefore, it may be regarded as a measure of learning (Venault et al. 1986).

### Grip-strength test

The effects of WIN, classical antiepileptic drugs and their combinations, at the ED_50_ values from the mouse 6 Hz test on skeletal muscular strength in mice were quantified by the grip-strength test of Meyer et al. (1979). The time before the commencement of the grip-strength test (after drug administration) was identical to that for the mouse 6 Hz test. The grip-strength apparatus (BioSeb, Chaville, France) comprised a wire grid (8 × 8 cm) connected to an isometric force transducer (dynamometer). The mice were lifted by the tails so that their forepaws could grasp the grid. The mice were then gently pulled backward by the tail until the grid was released. The maximal force exerted by the mouse before losing grip was recorded. The mean of three measurements for each animal was calculated and, subsequently, the mean maximal force of eight animals per group was determined. The skeletal muscular strength in mice was expressed in N (newtons) as mean ± SEM of eight determinations.

### Chimney test

The effects of WIN, classical antiepileptic drugs and their combinations, at the ED_50_ values from the mouse 6 Hz test on motor performance in mice were quantified with the chimney test of Boissier et al. (1960). The time before the commencement of the chimney test (after drug administration) was identical to that for the 6 Hz test. In the chimney test, animals had to climb backwards up a plastic tube (30 cm length, 3 cm inner diameter). Motor impairment was indicated by the inability of the animals to perform the test within 60 s. Results are presented as the percentage of the mice showing motor impairment.

### Statistical analysis

The ED_50_ values with their 95 % confidence limits were calculated by computer log-probit analysis according to Litchfield and Wilcoxon (1949). The obtained 95 % confidence limits were transformed to standard errors of the mean (SEM) as described previously (Luszczki et al. 2009). Microsoft’s Excel spreadsheet was used to perform the respective calculations. This spreadsheet was programmed to compute all calculations automatically and determine the dose–response relationship curves of the antiepileptic drugs administered alone and in combination with WIN from the log-probit linear regression analysis according to Litchfield and Wilcoxon (1949). Subsequently, the ED_50_ values were statistically analyzed using one-way analysis of variance (ANOVA) followed by the post hoc Tukey–Kramer test for multiple comparisons. Total brain antiepileptic drug concentrations were statistically compared using the unpaired Student’s *t* test. Qualitative variables from the chimney test were compared using Fisher’s exact probability test. The results obtained in the step-through passive avoidance task were statistically evaluated using Kruskal–Wallis non-parametric ANOVA followed by the post hoc Dunn’s multiple comparison test. The results from the grip-strength test were verified with one-way ANOVA followed by the post hoc Dunnett’s multiple comparison test. Differences among values were considered statistically significant if *P* < 0.05. All statistical tests were performed using commercially available GraphPad Prism version 4.0 for Windows (GraphPad Software, San Diego, CA, USA).

## Results

### Effect of WIN on the anticonvulsant activity of four classical antiepileptic drugs in the mouse 6 Hz psychomotor seizure model

All antiepileptic drugs studied, i.e., clonazepam, clobazam, phenobarbital and valproate displayed clear-cut anticonvulsant effects in the mouse 6 Hz-induced psychomotor seizure model. The ED_50_ values for all antiepileptic drugs calculated from their dose–response curves according to the log-probit method are presented in Table 1. WIN administered systemically (i.p.) at doses of 2.5 and 5 mg/kg had no significant impact on the anticonvulsant effects of clobazam in the 6 Hz test in mice (Table 1). In contrast, WIN 2.5 and 5 mg/kg significantly enhanced the anticonvulsant activity of clonazepam in the 6 Hz test by reducing its ED_50_ value by 68 % (*P* < 0.01) and 87 % (*P* < 0.001), respectively (Table 1). Only, WIN at 1.25 mg/kg had no significant impact on the anticonvulsant action of clonazepam in the mouse 6 Hz model (Table 1). Moreover, WIN at 5 mg/kg significantly potentiated the anticonvulsant action of phenobarbital and valproate, by reducing the ED_50_ value for phenobarbital by 49 % and for valproate by 47 %, respectively (*P* < 0.05; Table 1). WIN at a lower dose of 2.5 mg/kg had no significant effect on the anticonvulsant action of phenobarbital and valproate against 6 Hz-induced psychomotor seizures in mice (Table 1).

**Table 1:** Effect of WIN 55,212-2 mesylate on the protective activity of various classical antiepileptic drugs against 6 Hz-induced psychomotor seizures in mice

Treatment (mg/kg)	ED_50_ (mg/kg)	*n*
Clobazam + vehicle	1.96 ± 0.22	24
Clobazam + WIN (2.5)	1.62 ± 0.24	32
Clobazam + WIN (5)	1.58 ± 0.35	32
*F*(2,85) = 0.4734; *P* = 0.6245		
Clonazepam + vehicle	0.0164 ± 0.0030	24
Clonazepam + WIN (1.25)	0.0100 ± 0.0030	8
Clonazepam + WIN (2.5)	0.0052 ± 0.0012**	32
Clonazepam + WIN (5)	0.0022 ± 0.0008***	8
*F*(3,68) = 7.035; *P* = 0.0003		
Phenobarbital + vehicle	12.72 ± 1.73	24
Phenobarbital + WIN (2.5)	8.00 ± 1.50	32
Phenobarbital + WIN (5)	6.49 ± 1.51*	24
*F*(2,77) = 3.857; *P* = 0.0253		
Valproate + vehicle	116.69 ± 14.37	40
Valproate + WIN (2.5)	75.23 ± 15.10	32
Valproate + WIN (5)	62.11 ± 10.79*	24
*F*(2,93) = 4.106; *P* = 0.0196		

Results are presented as median effective doses (ED_50_ in mg/kg ± SEM) of antiepileptic drugs, protecting 50 % of animals tested against 6 Hz-induced seizures in mice. All antiepileptic drugs were administered i.p.: phenobarbital—60 min, clobazam and valproate—30 min and clonazepam—15 min prior to the 6 Hz-induced seizure test. WIN 55,212-2 mesylate was administered i.p. at 20 min before the 6 Hz-induced seizure test. Statistical analysis of the data was performed with log-probit method and one-way ANOVA followed by the post hoc Tukey–Kramer test for multiple comparisons. n—total number of animals used at those doses whose anticonvulsant effects ranged between four and six probits

* *P* < 0.05; ** *P* < 0.01; *** *P* < 0.001 versus control (antiepileptic drug + vehicle-treated) animals

### Effect of WIN on total brain antiepileptic drug concentrations

With fluorescent polarization immunoassay, total brain concentration of clonazepam administered alone at a dose of 2.2 mg/kg was 34.78 ± 2.02 ng/ml and did not differ significantly from that determined when clonazepam (2.2 mg/kg) was administered in combination with WIN (5.0 mg/kg), which amounted to 35.19 ± 2.18 ng/ml. Because the analytical technique employed to quantify clonazepam concentrations at a dose of 0.0022 mg/kg was not sensitive enough, the drug was evaluated at a dose of 2.2 mg/kg (i.e., 1,000-fold higher). Total brain concentration of phenobarbital administered alone at a dose of 6.49 mg/kg was 4.07 ± 0.12 μg/ml and did not differ significantly from that determined for the antiepileptic drug (6.49 mg/kg) in combination with WIN (5.0 mg/kg), which was 4.03 ± 0.18 μg/ml. Similarly, total brain concentration of valproate administered alone at a dose of 62.11 mg/kg was 44.91 ± 4.55 μg/ml and that of valproate (62.11 mg/kg) in combination with WIN (5.0 mg/kg) was 48.32 ± 4.67 μg/ml, indicating no significant difference between these concentrations with unpaired Student’s *t*-test.

### Influence of the antiepileptic drugs administered in combinations with WIN on motor performance, learning and skeletal muscular strength in the chimney, passive avoidance and grip-strength tests

In case of WIN administered alone at the dose of 5 mg/kg, it was found that the non-specific cannabinoid CB_1_ and CB_2_ receptor agonist had no significant impact on motor coordination in the chimney test, learning in the passive avoidance task or skeletal muscular strength in the grip-strength test in mice (Table 2). When WIN (5 mg/kg) was administered in combination with clobazam, clonazepam, phenobarbital and valproate (at doses corresponding to their ED_50_ values from the 6 Hz test), it did not impair motor coordination or learning or affect muscular skeletal strength as assessed by the chimney test, passive avoidance task and grip-strength test, respectively (Table 2).

**Table 2:** Effects of WIN 55,212-2 mesylate in combinations with four classical antiepileptic drugs on learning, muscular strength and motor performance in mice

Treatment (mg/kg)	Retention time (*s*)	Grip-strength (*N*)	Motor coordination impairment (%)
Vehicle	180 (180; 180)	0.92 ± 0.04	0
WIN (5.0) + vehicle	173.5 (155; 180)	0.91 ± 0.04	25
Clobazam (1.58) + WIN (5.0)	180 (180; 180)	0.90 ± 0.05	12.5
Clonazepam (0.0022) + WIN (5.0)	180 (180; 180)	0.91 ± 0.05	0
Phenobarbital (6.49) + WIN (5.0)	180 (180; 180)	0.90 ± 0.04	12.5
Valproate (62.11) + WIN (5.0)	180 (155.8; 180)	0.90 ± 0.05	25

Results are presented as: (1) median retention times (in seconds; with 25th and 75th percentiles in parentheses) from the passive avoidance task, assessing learning in mice; (2) mean grip-strengths (in Newtons ± SEM) from the grip-strength test, assessing skeletal muscular strength in mice; and (3) percentage of animals showing motor coordination impairment in the chimney test in mice. Each experimental group consisted of eight mice. Statistical analysis of the data from the passive avoidance task was performed with non-parametric Kruskal–Wallis ANOVA test. Results from the grip-strength test were analyzed with one-way ANOVA. The Fisher’s exact probability test was used to analyze the results from the chimney test. All drugs were administered i.p. at specific pretreatment times scheduled from the 6 Hz-induced psychomotor seizures and at doses corresponding to their ED_50_ values against 6 Hz-induced convulsions in mice (for more details see the legend to Table 1)

## Discussion

Results presented herein indicate that WIN enhanced the anticonvulsant action of clonazepam, phenobarbital and valproate, but not that of clobazam in the mouse 6 Hz-induced psychomotor seizure test. The selection of classical antiepileptic drugs (i.e., clobazam, clonazepam, phenobarbital and valproate) in this study was based on information that these antiepileptic drugs suppressed, in a dose dependent manner, the 6 Hz-induced psychomotor seizures in mice (Barton et al. 2001; Rowley and White 2010; Smith et al. 2007). In contrast, some classical and second-generation antiepileptic drugs, i.e., carbamazepine, phenytoin, topiramate and vigabatrin, are virtually ineffective in this seizure model (Florek-Luszczki et al. 2014) and, therefore, these antiepileptic drugs were not tested in the presented study.

It is worth mentioning that WIN administered alone at doses up to 5 mg/kg did not protect any animals against 6 Hz-induced psychomotor seizures. This is the reason that WIN was administered in the presented study at doses up to 5 mg/kg when combined with each of the selected classical antiepileptic drugs.

The results presented herein confirm our previous observations showing that WIN significantly enhanced the anticonvulsant action of phenobarbital and valproate (Table 3; Luszczki et al. 2011a, b). Previously, it has been documented that WIN at 15 mg/kg significantly potentiated the anticonvulsant action of phenobarbital and valproate in the mouse PTZ-induced seizure test (Table 3; Luszczki et al. 2011a). Unfortunately, WIN at lower doses of 5 and 10 mg/kg had no impact on the anticonvulsant action of phenobarbital and valproate in the mouse PTZ-induced clonic seizure model (Luszczki et al. 2011a). In the mouse MES model, WIN 10 mg/kg significantly potentiated the anticonvulsant action of phenobarbital and valproate. Similarly, WIN 5 mg/kg also potentiated the antiseizure action of valproate, but not that of phenobarbital in the mouse MES model (Table 3; Luszczki et al. 2011b). In the presented study, WIN 5 mg/kg significantly enhanced the anticonvulsant action of phenobarbital and valproate in the mouse 6 Hz-induced psychomotor seizure model. In contrast, WIN at a dose of 2.5 mg/kg had no impact on the anticonvulsant action of phenobarbital and valproate in the 6 Hz model.

**Table 3:** Influence of WIN 55,212-2 mesylate on the anticonvulsant action of the studied antiepileptic drugs in various animal seizure models

	Seizure model		
Antiepileptic drug	MES	PTZ	6 Hz
Clobazam	↑ (2.5 and 5 mg/kg)^a^	N.T.	0 (5 mg/kg)^d^
Clonazepam	N.T.	0 (15 mg/kg)^c^	↑ (2.5 and 5 mg/kg)^d^
Phenobarbital	↑ (10 mg/kg)^b^	↑ (15 mg/kg)^c^	↑ (5 mg/kg)^d^
Valproate	↑ (10 mg/kg)^b^	↑ (15 mg/kg)^c^	↑ (5 mg/kg)^d^

Doses of WIN, which significantly potentiated the anticonvulsant activity of the studied antiepileptic drugs, are given in parentheses

MES—maximal electroshock-induced seizure test, PTZ—pentylenetetrazole-induced seizure test, 6 Hz—psychomotor (limbic) 6 Hz-induced seizure test, ↑—increase in the anticonvulsant activity of the studied antiepileptic drug, 0—no significant effect despite the administration of WIN at a maximally tested dose, N.T.—not tested

^a^unpublished data

^b^Results from Luszczki et al. 2011b

^c^Results from Luszczki et al. 2011a

^d^Results from this study

Moreover, it was documented herein that WIN at doses of 2.5 and 5 mg/kg significantly potentiated the anticonvulsant action of clonazepam in the mouse 6 Hz psychomotor seizure test. In contrast, WIN (at doses up to 15 mg/kg) did not affect the anticonvulsant action of clonazepam in the mouse PTZ-induced clonic seizure model (Table 3; Luszczki et al. 2011a). The observed discrepancy could be explained through different seizure models used. Since molecular mechanisms of action of clonazepam and WIN were the same in both 6 Hz and PTZ tests, the apparent difference in the antiseizure activity of clonazepam after administration of WIN must result from diverse induction of seizure activity in animals (i.e., electrically vs. chemically evoked seizures).

In the case of clobazam, WIN did not significantly affect the anticonvulsant action of the antiepileptic drug in the 6 Hz model. In contrast, WIN 2.5 and 5 mg/kg significantly enhanced the anticonvulsant action of clobazam in the mouse MES model (unpublished data, Table 3). Similarly, since molecular mechanisms of action of clobazam and WIN were the same in both 6 Hz and MES tests, the apparent difference in the antiseizure activity of clobazam after administration of WIN must result from diverse induction of seizure activity in animals (i.e., electrically evoked seizures).

Of note, a substantial difference in pharmacological action of clonazepam and clobazam after administration of WIN in the 6 Hz model was observed. Considering molecular mechanisms of action of clobazam and clonazepam, it can be ascertained that both drugs substantially differ from one another. Although, both antiepileptic drugs are benzodiazepines and both evoke GABA_A_ receptor-mediated suppression of seizures, clonazepam and clobazam must differ in relation to their intrinsic activity on GABA_A_ receptors after WIN administration. Bearing in mind a substantial difference in the action of clonazepam and clobazam after co-administration of WIN in the mouse 6 Hz model, one can suggest that clonazepam is more active than clobazam in this seizure model (Table 3). On comparing results presented in Table 3, one can ascertain that WIN potentiated the anticonvulsant action of clonazepam in the 6 Hz model and clobazam in the mouse MES model, remaining almost inactive when combined with clonazepam in the mouse PTZ model and with clobazam in the 6 Hz model. Thus, the influence of WIN seems to be specific for seizure model used in experimental studies.

Of note, clobazam (7-chloro-1-methyl-5-phenyl-1,5-benzodiazepine-2,4-dione), in contrast to clonazepam (5-(2-chlorophenyl)-7-nitro-1,3-dihydro-1,4-benzodiazepin-2-one), has a 1,5 substitution instead of the usual 1,4-benzodiazepine structure, which results in a reduction of the sedative effects without losing its anticonvulsant effects (Schmidt 2002). Generally, clobazam is better tolerated than other benzodiazepines. This is the reason for clobazam being used as an excellent second-line therapy in some patients with resistant epilepsy (Schmidt 2002). With regard to clobazam, it enhances GABAergic activity by binding to the α subunit of the GABA_A_ receptor and increasing the frequency of chloride channel conductance by allosteric activation of the GABA_A_ receptor (Ng and Collins 2007). Moreover, clobazam increases expression of glutamate transporter protein 1 (GLT1) and GABA transporter protein 3 (GAT3) in the brain (Doi et al. 2005). In case of clonazepam, the drug as a benzodiazepine works by primarily enhancing GABAergic inhibition by binding to the benzodiazepine receptor on GABA_A_ receptors (Patsalos 2005).

The apparent difference in the anticonvulsant action of clobazam and clonazepam after WIN administration in the 6 Hz seizure model may result from their different affinity to benzodiazepine receptors in the brain. Although this hypothesis is highly speculative, it can readily explain the observed difference in the anticonvulsant activity of the drugs after administration of WIN. To confirm or reject this hypothesis, more advanced molecular studies are required.

It is highly likely that WIN-related activation of cannabinoid CB_1_ and CB_2_ receptors potentiated clonazepam-induced activation of GABA_A_ receptors in the brains of experimental animals subjected to 6 Hz-induced psychomotor (limbic) seizures, and thus, a favorable combination by suppressing 6 Hz-induced psychomotor seizures in mice can be reported. With regard to WIN and its interaction with GABA_A_ receptors, it has been found in in vitro studies that WIN decreased the cumulative amplitude of inhibitory postsynaptic currents (IPSCs) with maximal inhibition observed at 20 min after WIN administration (Kovacs et al. 2012). WIN inhibited GABAergic synaptic transmission by activating cannabinoid CB_1_ receptors at the presynaptic axon terminals and did not modify the effect of released GABA on the postsynaptic neurons (Kovacs et al. 2012). In contrast, AM251 (1-(2,4-dichlorophenyl)-5-(4-iodophenyl)-4-methyl-*N*-(1-piperidyl)pyrazole-3-carboxamide—a cannabinoid CB_1_ receptor antagonist) and rimonabant (5-(4-chlorophenyl)-1-(2,4-dichlorophenyl)-4-methyl-*N*-(piperidin-1-yl)-1*H*-pyrazole-3-carboxamide—a cannabinoid CB_1_ receptor inverse agonist) allosterically potentiate GABA_A_ receptors expressed in *Xenopus oocytes* at nM concentrations (Baur et al. 2012). The site of action of AM251 and rimonabant is not identical to that of benzodiazepines, loreclezole, phenobarbital or neurosteroids (Baur et al. 2012). Experimental evidence indicates that the endogenous cannabinoid 2-arachidonoyl glycerol potentiates GABA_A_ receptors containing β_2_ subunit, at low concentrations of GABA (Sigel et al. 2011, Baur et al. 2013). Since 2-arachidonoyl glycerol is biosynthesized in postsynaptic neurons, the endocannabinoid interacts locally with GABA_A_ receptors within the postsynaptic neurons (Sigel et al. 2011). Additionally, it has been documented in in vitro study that 2-arachidonoyl glycerol interacted supra-additively (synergistically) with 3α,21-dihydroxy-5α-pregnan-20-one (a neurosteroid) and diazepam (a benzodiazepine), suggesting that this endocannabinoid modulates the action of neurosteroids and benzodiazepines at extra-synaptic and synaptic β_2_ subunit-containing GABA_A_ receptors (Sigel et al. 2011). Considering the above-discussed facts, it is highly likely that WIN, despite the inhibition of GABAergic synaptic transmission at presynaptic neuronal terminals, enhances response of GABA_A_ receptors in postsynaptic neurons. Since supra-additive interaction has been documented in vitro between 2-arachidonoyl glycerol (an endogenous cannabinoid) and diazepam (a benzodiazepine), it is highly likely that WIN can also supra-additively interact with clonazepam in in vivo experiments.

With regard to acute adverse effects produced by the antiepileptic drugs in combination with WIN, it can be ascertained that all the studied antiepileptic drugs administered either alone or in combination with WIN (at doses corresponding to their ED_50_ values from the 6 Hz test) did not exert acute adverse effects as determined in the chimney, passive avoidance and grip-strength tests in mice. Previously, we reported that WIN administered either alone or in combination with various antiepileptic drugs (at doses corresponding to the ED_50_ values of the tested antiepileptic drugs) significantly impaired motor coordination in the chimney test, disturbed learning processes in the passive avoidance task and alleviated muscular strength in the grip-strength test in mice (Luszczki et al. 2011a, b). More specifically, it has been documented that WIN administered alone at a dose of 10 mg/kg significantly reduced skeletal muscular strength in mice subjected to the grip-strength test (Luszczki et al. 2011b). Likewise, WIN (10 mg/kg) combined with carbamazepine, phenobarbital, phenytoin and valproate (at doses corresponding to the ED_50_ values from the MES test) considerably reduced skeletal muscular strength in mice (Luszczki et al. 2011b). In the step-through passive avoidance task, WIN combined with phenobarbital, phenytoin and valproate significantly disturbed learning in mice (Luszczki et al. 2011b). In the chimney test, WIN combined with phenobarbital and valproate significantly impaired motor performance in mice (Luszczki et al. 2011b). Similar results were observed when WIN (15 mg/kg) was combined with clonazepam, ethosuximide, phenobarbital and valproate. More specifically, all combinations of WIN (15 mg/kg) with clonazepam, ethosuximide, phenobarbital and valproate (at doses corresponding to the ED_50_ values from the PTZ-induced seizure test) significantly impaired learning in the passive avoidance task, reduced skeletal muscular strength in the grip-strength test and impaired motor coordination in the chimney test (Luszczki et al. 2011a). However, in the present study, no significant changes in motor performance, learning and muscular strength were observed in mice because of a low dose of WIN used (5 mg/kg). Of note, the observed slight impairment of motor coordination in mice receiving WIN alone or WIN in combination with the studied antiepileptic drugs (up to 25 %) was not significant with the Fisher’s exact probability test. Thus, the combinations of WIN with the tested antiepileptic drugs are worthy of consideration when used in patients.

Moreover, pharmacokinetic evaluation of total brain antiepileptic drugs concentrations revealed that WIN 5 mg/kg did not affect total brain concentrations of clonazepam, phenobarbital and valproate in experimental animals. Our pharmacokinetic study is consistent with that documenting earlier that WIN did not affect total brain concentrations of phenobarbital and valproate in mice (Luszczki et al. 2011a, b). On the other hand, because there were no pharmacokinetic changes in total brain concentrations of clonazepam, phenobarbital and valproate after co-administration of WIN, it can be ascertained that the enhanced anticonvulsant action of clonazepam, phenobarbital and valproate in the 6 Hz model was a result of pharmacodynamic interactions between the tested drugs. Because WIN did not significantly affect the protective action of clobazam against 6 Hz-induced psychomotor seizures, we did not estimate the total brain concentrations of the antiepileptic drug in mice. However, it can be assumed that since WIN did not affect total brain concentrations of clonazepam, phenobarbital and valproate in mice, the non-selective cannabinoid CB_1_ and CB_2_ receptor agonist (WIN) would not affect total brain concentrations of clobazam.

## Conclusions

Based on this preclinical study, it can be concluded that the combinations of WIN with clonazepam, phenobarbital and valproate can potentially offer patients with limbic seizures favorable combinations and worthy of clinical evaluation. In all cases, because a substantial dose reduction of antiepileptic drugs in the mixture can be anticipated, it can be expected that concurrent adverse effects would be significantly reduced and this is a clinically desirable outcome. Since WIN had no impact on clobazam’s anticonvulsant action in the 6 Hz model, this combination should not be recommended for patients with epilepsy. If the results from this study could be extrapolated into clinical settings, a novel therapeutic option in the treatment of limbic epilepsy would be created.

## Abbreviations

MES: Maximal electroshock-induced seizures
PTZ: Pentylenetetrazole-induced seizures
WIN: WIN 55,212-2
